# Potential of Native Rhizobia in Enhancing Nitrogen Fixation and Yields of Climbing Beans (*Phaseolus vulgaris* L.) in Contrasting Environments of Eastern Kenya

**DOI:** 10.3389/fpls.2017.00443

**Published:** 2017-03-31

**Authors:** Gilbert Koskey, Simon W. Mburu, Ezekiel M. Njeru, Jacinta M. Kimiti, Omwoyo Ombori, John M. Maingi

**Affiliations:** ^1^Department of Microbiology, Kenyatta UniversityNairobi, Kenya; ^2^Department of Forestry and Land Resources Management, South Eastern Kenya UniversityKitui, Kenya; ^3^Department of Plant Sciences, Kenyatta UniversityNairobi, Kenya

**Keywords:** native rhizobia, ecosystem services, biological nitrogen fixation, climbing beans, Eastern Kenya

## Abstract

Climbing bean (*Phaseolus vulgaris* L.) production in Kenya is greatly undermined by low soil fertility, especially in agriculturally prolific areas. The use of effective native rhizobia inoculants to promote nitrogen fixation could be beneficial in climbing bean production. In this study, we carried out greenhouse and field experiments to evaluate symbiotic efficiency, compare the effect of native rhizobia and commercial inoculant on nodulation, growth and yield parameters of mid-altitude climbing bean (MAC 13 and MAC 64) varieties. The greenhouse experiment included nine native rhizobia isolates, a consortium of native isolates, commercial inoculant Biofix, a mixture of native isolates + Biofix, nitrogen treated control and a non-inoculated control. In the field experiments, the treatments included the best effective native rhizobia isolate ELM3, a consortium of native isolates, a commercial inoculant Biofix, a mixture of native isolates + Biofix, and a non-inoculated control. Remarkably, four native rhizobia isolates ELM3, ELM4, ELM5, and ELM8 showed higher symbiotic efficiencies compared to the Biofix. Interestingly, there was no significant difference in symbiotic efficiency between the two climbing bean varieties. Field results demonstrated a significant improvement in nodule dry weight and seed yields of MAC 13 and MAC 64 climbing bean varieties upon rhizobia inoculation when compared to the non-inoculated controls. Inoculation with ELM3 isolate resulted to the highest seed yield of 4,397.75 kg ha^−1^, indicating 89% increase over non-inoculated control (2,334.81 kg ha^−1^) and 30% increase over Biofix (3,698.79 kg ha^−1^). Farm site significantly influenced nodule dry weight and seed yields. This study, therefore, revealed the potential of native rhizobia isolates to enhance delivery of agroecosystem services including nitrogen fixation and bean production. Further characterization and mapping of the native isolates will be imperative in development of effective and affordable commercial inoculants.

## Introduction

Climbing bean (*Phaseolus vulgaris* L.) is one of the most important food crops that is widely cultivated in Sub-Saharan Africa (SSA) and other tropical regions (Ramaekers et al., 2013). The crop is a short-season legume with most varieties maturing between 65 and 110 days after emergence. Production of climbing beans by smallholder farmers is often constrained by the impoverished soil fertility, poor agronomic practices, bean diseases and pest infestation, thus resulting in minimal yields (Beebe et al., 2012). The increasing human population in SSA has led to fragmentation and intensive use of agriculturally prolific lands, leading to exhaustion of available soil nutrients. For instance, each household in Eastern Kenya is estimated to have an average of 0.5–1.0 hectares agriculturally productive farm (Mburu et al., 2016). Limited soil nitrogen has been pointed out as one of the restraining factors in bean production (Shamseldin et al., 2012). To counter soil nitrogen limitation, organic manure, inorganic nitrogen fertilizers and bio-fertilizers are used. However, organic manure is rarely available for smallholder farmers (Gichangi et al., 2012). On the other hand, inorganic nitrogen fertilizers, which boost bean production, are costly and unaffordable to the resource poor smallholder farmers. In addition, the use of inorganic fertilizers has drawn a number of reactions due to negative environmental effects especially on soil biodiversity and aquatic ecosystems (Hester and Harrison, 2012; Mutuma et al., 2014).

Biological nitrogen fixation (BNF) has been widely used as a replacement of nitrogen fertilizers in legume production because of its economic efficiency in the provision of sustainable agroecosystem services (Ouma et al., 2016). Rhizobia are nitrogen-fixing bacteria that live either freely in the soil or form a symbiotic association with the roots of legumes (Martínez-Romero, 2003). Rhizobia are used as bio-fertilizers in legume production and are reported to increase the availability of nitrogen through BNF in different agroecosystems, hence enhancing plant growth and yields (Chabot et al., 1998). Rhizobia biofertilizers are of economic importance in climbing bean production since they offer an alternative farming technology that is eco-friendly, sustainable and enhances soil biodiversity and soil structure (Rahmani et al., 2011). The utilization of native rhizobia as inoculants promote ecologically sustainable management of agricultural ecosystems and enhance legume production due to their growth promoting traits and adaptability to soil and environmental stress (Mwangi et al., 2011). Furthermore, crop production using inoculants could be cheaper and more affordable to the resource-poor smallholder farmers (Singh et al., 2016). The ability of native strains to interact positively with the resident soil microbiota and their adaptability to the local agroecological climatic conditions often elucidates their superior performance over the exotic commercial strains (Meghvansi et al., 2010).

Despite the beneficial effects of rhizobia biofertilizers, it is often necessary to provide legumes with rhizobia inoculants that are infective, and effective in causing nodulation and nitrogen fixation (Tena et al., 2016). Strain screening and selection is an important step in inoculum development. The existence of native rhizobia isolates that successfully nodulate legumes has been demonstrated in different parts of the world (Anyango et al., 1995; Rahmani et al., 2011; Stajković et al., 2011). Recently, species of the genus *Rhizobium*, such as *R. etli, R. giardinii, R. leguminosarum*, and *R. tropici* have been reported to nodulate and establish a symbiotic association with different common bean varieties in tropical areas (Torres et al., 2009; Ribeiro et al., 2013). In Kenya, Mathu et al. (2012) demonstrated the potential of native bradyrhizobia to improve cowpea and green gram yields. Variable responses to inoculation using commercial inoculants have been reported and this highlights the need to identify specific native rhizobia strains or their combinations for legume production. The use of host-specific native rhizobia isolates is recommended because they adapt better to the local environmental and soil conditions (Ouma et al., 2016). In addition, native rhizobia isolates are persistent and have better survival rate (Stajković et al., 2011) and this could increase the chances of successful nodulation and nitrogen fixation in the host plant. On the other hand, the inability of introduced commercial inoculants to compete well with native rhizobia population due to negative microbial interactions impedes their use (Martínez-Romero, 2003).

Authentication of rhizobia to determine their symbiotic efficiency is required to screen out effective native rhizobia isolates. This is usually carried out in a greenhouse under bacteriologically controlled conditions (Beck et al., 1993; Maingi et al., 2001). In order to achieve maximum legume productivity, screening of native isolates for their nitrogen fixation efficiencies is vital (Anglade et al., 2015). Furthermore, screening is important in the development of effective legume inoculum. Limited information on the symbiotic nitrogen-fixing potential of the mid-altitude climbers (MAC) with native rhizobia isolates in contrasting environments of Eastern Kenya is available. Previous studies have majorly focused on nitrogen fixation potential of bush beans, and traditional climbing bean varieties and their yield performance in different cropping conditions (Kimani et al., 2007; Gicharu et al., 2013; Ouma et al., 2016). The results from this study will contribute toward the development of rhizobia inoculum for use in the production of common beans in different agroecological zones. Moreover, the assessment of native rhizobia and their compatibility with the new bean lines, such as MAC varieties would contribute to the worldwide knowledge of soil microorganisms and their importance in BNF. There is need to identify native rhizobia isolates that nodulate with MAC beans and evaluate their symbiotic efficiency in the greenhouse and their suitability for use as inoculants in the field. The objectives of this study were to evaluate the symbiotic efficiency of native rhizobia isolates and to compare the effect of native rhizobia and commercial inoculants on nodulation, growth and yield parameters of MAC 13 and MAC 64 climbing bean varieties grown in different agroecological zones of Embu and Tharaka Nithi Counties in Eastern Kenya.

## Materials and methods

### Study sites

Greenhouse experiments were carried out in the Department of Microbiology, Kenyatta University in Nairobi, Kenya (1°11′10″S, 36°55′30″E). Field experiments were conducted in four selected farms in upper and lower midland agro-ecological zones of Embu and Tharaka Nithi Counties in Eastern Kenya. The experiments were carried out during the long rainy season (March to August 2015) and short rainy season (October to December 2015). The two farms in Embu County (ELM and EUM) are situated at the foot of Mt. Kenya at 0.53° S, 37.45° E within an elevation of 1100 to 1500 m above sea level (a.s.l) (Jaetzold et al., 2006). The area receives bimodal rainfall pattern with an average of 1500 mm annually. In Tharaka Nithi County, the two farms selected (TLM and TUM) are located on the South-Eastern side of Mt. Kenya at 0.30° S, 38.06° E and lie within an elevation of 600 m to 1500 m a.s.l. (Jaetzold et al., 2006). Tharaka Nithi County receives a relatively lower annual precipitation averaging at 1000 mm annually. The field experimental sites were selected based on agro-climatic conditions and prevalence of climbing bean cultivation. From the two Counties, the farms chosen had no history of rhizobia inoculation. The farm sizes had a characteristic of smallholder farming systems with an average of 0.5–1.0 Ha per household. The dominant crop species in the study area are common beans, maize, cowpeas, soybeans, bananas, tea, and coffee (Mburu et al., 2016). Climbing bean (*Phaseolus vulgaris* L.) varieties (MAC 13-Kenya safi and MAC 64-Kenya mavuno) were used as test plants. They are not only high yielding bean varieties but also heat tolerant and resistant to common bean diseases. The MAC 13 (cream white background with red flecks seeds) and MAC 64 (dark red mottled medium seeded) are suitable for production in an altitude range of 1000–1800 m asl (Ramaekers et al., 2013). The two bean varieties are commercially sold by the Kenya Seed Company Limited (Nairobi).

### Soil sampling and analyses

Soil sampling was carried out in all the four farms before the onset of long rains (March 2015). The soil was sampled across and diagonally from 20 points in each farm at a depth of 5–20 cm using a hand shovel. A kilogram of a homogeneous composite soil sample was made from each farm and packed independently into sterile bags for laboratory analysis. Soil samples were air-dried and sieved through a 2 mm diameter sieve for physical and chemical analysis. Soil analysis was carried out according to the procedures described by Okalebo et al. (2002).

### Rhizobia trap cultures

Field trapping of native rhizobia was carried out in all the four farms in Embu and Tharaka Nithi Counties from March to August 2015 using MAC 13 and MAC 64 climbing bean varieties obtained from Kenya Seed Company Limited, Nairobi. The farms were demarcated and prepared for planting by plowing and fine harrowing before the onset of the first rains. Two climbing bean seeds were planted per hill at a spacing of 75 cm by 30 cm. Triple superphosphate (TSP) fertilizers were applied at a rate of 46 kg P_2_O_5_ ha^−1^. During the mid-flowering stage, 10 plants from each farm were randomly sampled and harvested by carefully excavating the root systems to recover root nodules. The root nodules showing pink coloration from each bean plant were collected, packed in sterile sampling vials containing cotton wool and silica gel for desiccation. The nodules were transported to the Microbiology laboratory at Kenyatta University where they were air-dried for rhizobia isolation and storage.

### Isolation of native rhizobia from nodules

Air-dried nodules collected from MAC 13 and MAC 64 climbing beans were immersed in water and left to imbibe the water for 2 h. They were thoroughly washed and surface sterilized with 70% ethanol for 2 min and 3% sodium hypochlorite solution for 3 min (Somasegaran and Hoben, 1994). The sterile nodules were rinsed in seven changes of sterile distilled water and crushed in sterile universal bottles using a sterilized glass rod in normal saline solution. A loop-full of the resulting suspension was streaked on the surface of petri-dishes containing Yeast Extract Mannitol Agar (YEMA) medium supplemented with Congo red (0.0025% w/v). The streaked media were incubated in the dark at 28°C for 3–5 days (Vincent, 1970). The YEMA medium was prepared by dissolving 0.1 g NaCl; 10.0 g mannitol; 0.2 g MgSO_4_.7H_2_O; 0.5 g CaCO_3_; 0.5 g yeast extract; 15.0 g agar and 0.002 M FeCl_2_.6H_2_O in 1 l of distilled water. Emerging single colonies, which were typical of rhizobia species, were sub-cultured by repeated streaking on YEMA and YEMA containing bromothymol blue (BTB) (0.0025% w/v) plates. Based on morpho-cultural and biochemical characteristics, the isolates were placed into nine different groups and a representative isolate from each group was used in symbiotic efficiency test. Pure cultures were preserved on YEMA agar slants in screw-capped McCartney bottles for future use at 4°C.

### Greenhouse experiments

The greenhouse experiments were carried out to assess infectivity and symbiotic efficiency of native rhizobia isolates on MAC 13 and MAC 64 climbing bean varieties under controlled conditions. The crops were grown in an Even-span greenhouse (polyethylene roofing) with the following conditions; natural lighting of 12-h day/night, temperatures of 20–25°C and relative humidity between 60 and 65%.

### Authentication and symbiotic properties of the native rhizobia isolates

Climbing bean seeds of high quality (undamaged) were surface sterilized for 30 s using 70% alcohol, followed by 3% sodium hypochlorite solution for 3 min and they were finally rinsed in seven changes of sterile distilled water. Twenty seeds were cultured in each petri-plate containing water agar (10% agar in distilled water) and pre-germinated aseptically in the dark at 28°C for 3 days. Only 2 actively sprouting seedlings with radicles of length 1–2 cm were transferred aseptically into each modified Leonard jars containing sterile vermiculite (rooting medium) and nitrogen-free nutrient solutions (Broughton and Dilworth, 1971) covered with sterile aluminum foil.

Inoculation of seedlings was carried out after 8 days by pipetting 1 ml (10^9^ cfu/ml) of the respective rhizobia isolates into the root radical base. The greenhouse experiment was laid out in a completely randomized design (CRD) with 4 replicates per treatment. The treatments included; the representative native rhizobia isolates (ELM1, TUM2, ELM3, ELM4, ELM5, EUM6, EUM7, ELM8, and ELM9), native rhizobia consortium (TC), commercial rhizobia inoculant Biofix (TB) and a combination of all native rhizobia isolates + Biofix (TCB). Non-inoculated nitrogen treated (TN) and nitrogen-free (TUC) controls were included. The commercial rhizobia inoculant Biofix was supplied by MEA Company Limited, Nakuru, Kenya. The TN plants were constantly supplied with N (0.05% KNO_3_ w/v) solution. The plants were grown for 45 days, regularly adding N-free nutrient solution as required. They were harvested for nodulation, dry weight biomass and shoot nitrogen and phosphorus determination. Shoot nitrogen was analyzed using Kjeldahl method (Bremner, 1996), while shoot phosphorus concentration was determined using the photometric method after sulfuric-perchloric acid digestion (Njeru et al., 2014). Symbiotic efficiency (SEF%) was determined as previously described by Gibson (1987) and Beck et al. (1993); SEF (%) = Shoot dry weight (SDW) of inoculated plants/SDW of non-inoculated control plants supplemented with nitrogen (0.05% KNO_3_) and then converted into a percentage. The SEF values were rated as: >80% = highly effective, 51–80% = effective, 35–50% = lowly effective and <35% = ineffective (Lalande et al., 1990).

### Field experiments

Field experiments were carried out to compare the effect of native rhizobia and commercial inoculants on nodulation, growth and seed yield of MAC 13 and MAC 64 climbing bean varieties. The experiments were laid out in a randomized complete block design (RCBD) with three replications. Treatments included; the best native rhizobia isolate (ELM3), a consortium (TC) of all native isolates (ELM1, TUM2, ELM3, ELM4, ELM5, EUM6, EUM7, ELM8, and ELM9), commercial inoculant Biofix (TB) and a mixture of a consortium of native isolates + Biofix (TCB). Non-inoculated control (TUC) was included as a negative control. The plant spacing used was 75 cm by 30 cm. Each plot measured 3 × 3 m and a spacing of 1 m between the plots was left to minimize inter-plot interference.

### Land preparation and planting

The land was plowed and hand harrowed to a fine tilth before the first rain season (September 2015). A sterile filter mud was used as a carrier material for the rhizobia inoculants and inoculum was applied at recommended rate (100 g inoculum per 15 kg seeds) (Maingi et al., 2001). The Biofix commercial inoculum for beans was procured from MEA Company Limited and applied as per the manufacturer's instructions (100 g inoculum per 15 kg seeds). Only two climbing bean seeds of high quality were selected for planting. Seeds requiring rhizobia inoculation were prepared by coating with a filter mud containing respective inoculants using 4% gum Arabica (supplied with the inoculum). The negative control plots were left uninoculated and were planted a few hours before plots requiring inoculation in order to avoid cross contamination. Each treatment received a basal application of 46 kg P_2_O_5_ ha^−1^ (TSP) fertilizers during planting.

### Plant growth and harvesting

The climbing bean seedlings were thinned from two to one per hill 1 week after emergence. Weeds were controlled using hand hoeing over the growth period. During the mid-flowering stage, 3 plants from the central rows on each plot were selected randomly and harvested for assessment of nodulation and shoot biomass. The plant shoots were analyzed for nitrogen concentration (% N) using Kjeldahl method (Bremner, 1996), while shoot phosphorus was measured using photometry method after sulfuric-perchloric acid digestion (Njeru et al., 2014). At physiological maturity (after 95 days), 10 plants were randomly selected in each plot and manually harvested. Yield parameters per plant, such as the number of pods (NPP) and seed yield (SY) were assessed.

### Data analyses

The greenhouse and field data on nodule and shoot dry weight, shoot nitrogen and phosphorus, symbiotic efficiency, pod number and seed yield were subjected to analysis of variance (ANOVA) using Statistical Analysis Software (SAS) version 9.1. The means were separated using Tukey's HSD test at 5% significance level (Steel et al., 1997). Pearson correlation analysis was used to determine the association between nitrogen fixation parameters.

## Results

### Soil characteristics

The soil physical and chemical characteristics varied across the experimental sites in Embu and Tharaka Nithi Counties. The soils were characteristically acidic with pH ranging from 4.27 to 6.02 (Table 1). Soils from ELM had the highest organic carbon content (3.42%) and available phosphorus (32.15 ppm) while soil from EUM had the highest total nitrogen concentration (0.31%). The soil texture from EUM and TUM was classified as sandy clay, while soil from TLM and ELM were classified as clay and sandy clay loam, respectively (Table 1).

**Table 1:** **Soil characteristics of experimental study sites (before planting) compared with the critical values for East African soils**.

**Soil sample**	**pH**	**% O.C**	**% Total N**	**K (cmol/kg)**	**Available P (ppm)**	**% Sand**	**% Clay**	**% Silt**	**Texture class**
EUM	4.27	2.63	0.31	0.70	16.52	47	47	6	Sandy clay
ELM	6.02	3.42	0.22	0.85	32.15	54	27	19	Sandy clay loam
TUM	5.31	3.29	0.25	1.00	27.00	49	45	6	Sandy clay
TLM	5.85	3.33	0.20	1.80	25.10	45	49	6	Clay
^*^Critical value	5.50	3.00	0.25	0.22	15				

* *Okalebo et al. (2002). ELM, Embu Lower Midland; EUM, Embu Upper Midland; TLM, Tharaka Nithi Lower Midland; TUM, Tharaka Nithi Upper Midland; O.C, Organic Carbon; N, Nitrogen; K, Potassium; P, Phosphorus*.

### Morphological characteristics of the native rhizobia isolates

From this study, 9 distinct groups of isolates were obtained from the root nodules of MAC 13 and MAC 64 climbing beans grown during field trapping experiment in Eastern Kenya (Table 2). All isolates were identified as Gram-negative rods. The isolates did not absorb Congo red (CR) on streaking on YEMA-CR medium. There was no much variation observed in the colony shape, elevation and texture of the isolates. On YEMA-BTB medium, all isolates tested were acid producers and turned BTB indicator from deep green to yellow after 3 to 5 days of incubation in the dark. Out of the total 41 isolates obtained, group (iii) carried the highest percentage (28.89%), while group (ii) had the lowest percentage (2.22%) (Table 2).

**Table 2:** **Morpho-cultural characteristics of the rhizobia isolates trapped from the study farms**.

**Characteristic**	**Isolate grouping**								
	**i**	**ii**	**iii**	**iv**	**v**	**vi**	**vii**	**vii**	**ix**
CR absorption	Na	Na	Na	Na	Na	Na	Na	Na	Na
BTB reaction	Y	Y	Y	Y	Y	Y	Y	Y	Y
Gram reaction	-ve	-ve	-ve	-ve	-ve	-ve	-ve	-ve	-ve
Cell Shape	Rod	Rod	Rod	Rod	Rod	Rod	Rod	Rod	Rod
Elevation	Cvx	Cvx	Cvx	Cvx	Cvx	Cvx	Cvx	Raised	Cvx
Margin	Entire	Entire	Entire	Entire	Entire	Entire	Entire	Entire	Entire
Colony nature	Dull	Dull	Shiny	Shiny	Dull	Shiny	Shiny	Shiny	Shiny
Colony colour	Cy	Cw	Mw	Mw	Mw	Mw	W	Mw	Mw
Transparency	Op	Trl	Trl	Op	Op	Trl	Trl	Trl	Op
Colony dia. (mm)	1.5	3.5	1.0	3.0	0.5	3.5	5.0	1.0	1.0
Colony texture	Fg	Fg	Sm	Sm	Fg	Sm	Sm	Fg	Sm
Percentage %	4.44	2.22	28.89	6.67	8.89	20	17.78	4.44	6.67

*Na, Non-absorbing; Y, Yellow on BTB; -ve, Negative; Cvx, Convex elevation; Ent, Entire margin; Sny, Shiny; Cy, Cream yellow; Cw, Cream white; Mw, milky white; W, watery; Op, Opaque; Trl, Translucent; Fg, Firm gummy; Sm, Soft mucoid; Diam, Colony diameter*.

### Symbiotic efficiency of native rhizobia isolates on MAC 13 and MAC 64 climbing beans

**Figure 1:**
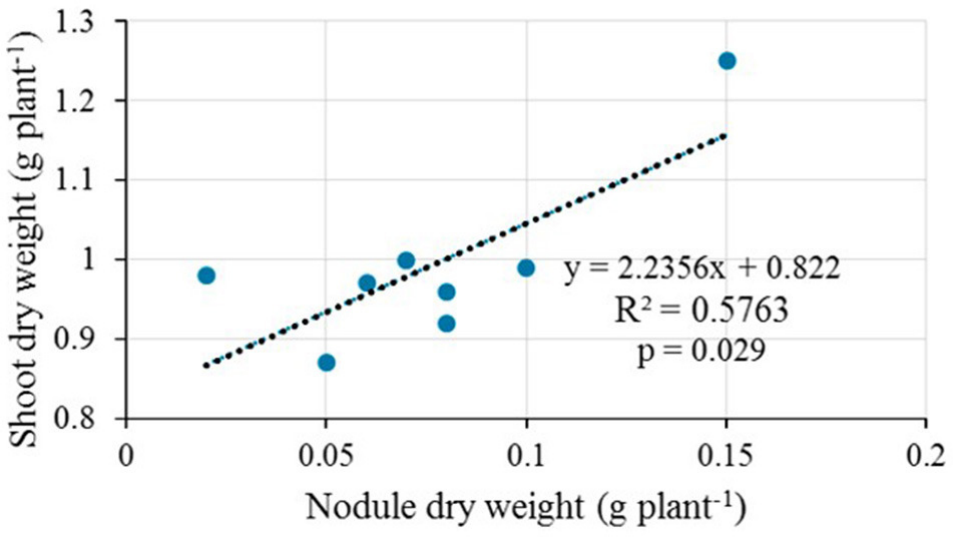
**Correlation analysis between nodule dry weight and shoot dry weight in the greenhouse**.

**Figure 2:**
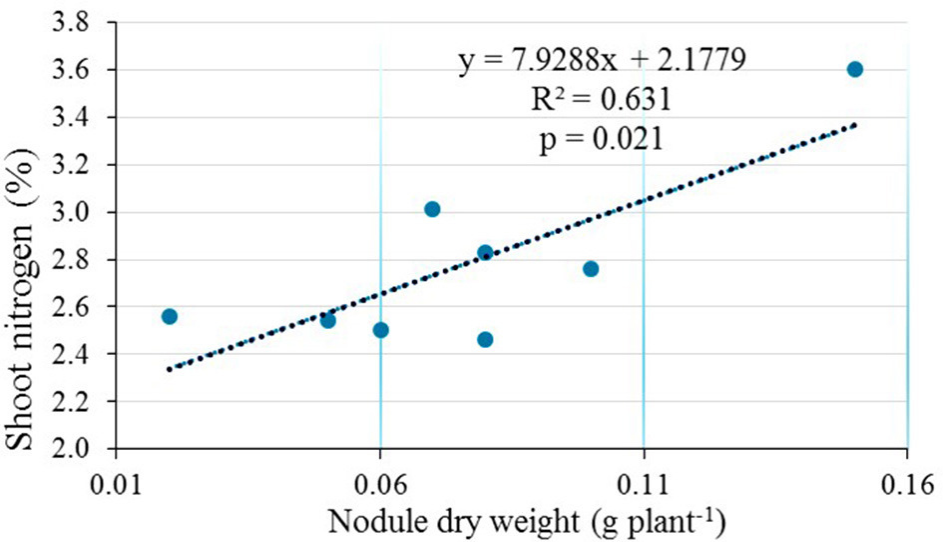
**Correlation analysis between nodule dry weight and shoot nitrogen in the greenhouse**.

Upon inoculation of test plants with 9 representative native isolates in the greenhouse, the isolates ELM3, ELM4, ELM5, ELM8, and ELM9 initiated nodulation and were thus, considered for symbiotic efficiency (SEF) determination. Native isolates ELM1, TUM2, EUM6, and EUM7 were not considered for SEF determination because they did not form nodules with the climbing beans. There was a significant positive effect of the rhizobia isolates on NDW (*p* < 0.001), SDW (*p* < 0.001), and SEF (*p* < 0.001) (Table 3). A significant (*p* < 0.001) improvement of all the tested parameters was observed in climbing beans inoculated with native isolate ELM3 compared to the commercial inoculant Biofix and the negative control. The effect of bean variety on NDW was statistically significant (*p* < 0.001) with MAC 64 having higher NDW compared to MAC 13 climbing bean variety. On the contrary, there was no significant difference between the two varieties in regard to % shoot N (*p* = 0.546), shoot phosphorus (*p* = 0.639), and SEF (*p* = 0.187) (Table 3). SEF values ranged from 86.17% in TC to 123.72% in native isolate ELM3. There was no significant interaction effect between bean variety and rhizobia isolates on NDW (*p* = 0.157) and SEF (*p* = 0.885). Correlation analysis showed a significant relationship between nodule dry weight and shoot dry weight of climbing beans (*R*^2^ = 0.5763, *p* = 0.029; Figure 1). Similarly, there was a strong positive correlation between nodule dry weight and shoot nitrogen (*R*^2^ = 0.631, *p* = 0.021; Figure 2).

**Table 3:** **Effect of rhizobia inoculation, bean variety, and their interaction on nodule dry weight, shoot dry weight, shoot nitrogen, and phosphorus and symbiotic efficiency in the greenhouse experiment**.

**Treatments**	**Nodule dry weight (NDW) g plant^−1^**	**Shoot dry weight (SDW) g plant^−1^**	**Shoot nitrogen (%N)**	**Shoot P (ppm)**	**Symbiotic efficiency (SEF) (%)**
**RHIZOBIA ISOLATES**					
ELM3	0.147 ± 0.016a	1.25 ± 0.07a	3.46 ± 0.11ab	3543.75 ± 184.36ab	123.72 ± 6.72a
ELM4	0.015 ± 0.008cd	0.98 ± 0.09abc	2.56 ± 0.20cd	3109.38 ± 208.51bc	96.75 ± 8.67abc
ELM5	0.072 ± 0.019bc	1.00 ± 0.05abc	3.01 ± 0.08bc	3234.38 ± 431.97b	99.21 ± 4.97abc
ELM8	0.095 ± 0.020ab	0.99 ± 0.06abc	2.56 ± 0.09cd	3488.75 ± 513.93ab	98.24 ± 5.26abc
ELM9	0.083 ± 0.019b	0.92 ± 0.03bcd	2.46 ± 0.10cd	3979.88 ± 116.28a	90.76 ± 3.02bcd
TC	0.054 ± 0.016bcd	0.87 ± 0.06bcd	2.44 ± 0.12cd	2623.75 ± 200.32de	86.17 ± 5.67bcd
TB	0.077 ± 0.091b	0.96 ± 0.07abc	2.83 ± 0.07bc	3476.00 ± 308.06ab	95.21 ± 7.31abc
TN	-	1.01 ± 0.06ab	3.71 ± 0.09a	2436.63 ± 110.69e	100.00 ± 5.43ab
TCB	0.063 ± 0.015bc	0.97 ± 0.4abc	2.11 ± 0.14d	1771.88 ± 8.44f	96.53 ± 3.87abc
TUC	-	0.71 ± 0.03cd	0.73 ± 0.02e	3087.50 ± 394.18c	-
**VARIETY**					
MAC 13	0.035 ± 0.01b	0.96 ± 0.03a	2.37 ± 0.12a	3012.36 ± 159.67a	92.77 ± 2.43a
MAC 64	0.051 ± 0.01a	0.87 ± 0.03b	2.32 ± 0.11a	3046.21 ± 99.73a	88.59 ± 3.03a
***P*-VALUES OF THE MAIN FACTORS AND THEIR INTERACTIONS**					
Rhizobia isolates	< 0.001	< 0.001	< 0.001	< 0.001	< 0.001
Variety	0.0147	0.011	0.546	0.639	0.187
Variety × Rhizobia isolates	0.157	0.912	0.861	0.510	0.885

*Means followed by same lower case letter(s) within the same column are not significantly different at p < 0.05 according to Tukey's HSD test. ELM3, ELM4, ELM5, ELM8 and ELM9, Native rhizobia isolates; TC, Consortium of native rhizobia; TB, Commercial inoculant (Biofix); TN, Negative control with nitrogen treatment; TCB, Biofix combined with consortium; TUC, Negative control without nitrogen; MAC, Mid altitude climbers. ELM, Embu Lower Midland*.

### Field experiments

#### Effect of rhizobia inoculation on nodulation and plant biomass

Rhizobia inoculation significantly enhanced NDW (*p* < 0.001) and SDW (*p* < 0.001) of climbing beans compared to non-inoculated controls (Table 4). Climbing beans inoculated with native isolate ELM3 had the highest NDW and SDW while non-inoculated beans had the lowest NDW and SDW (Table 4). The mean NDW and SDW of climbing beans inoculated with the native consortium, Biofix and a mixture of native consortium + Biofix were not statistically different. Between the two varieties, MAC 64 recorded a higher mean NDW of 0.09 g plant^−1^ compared to MAC 13 with a mean of 0.08 g plant^−1^. However, there was no significant (*p* = 0.095) difference in SDW of the two bean varieties. The climbing bean NDW and SDW varied significantly (*p* < 0.001) in all the farm sites with climbing beans from ELM recording the highest NDW and SDW while climbing beans grown in EUM recorded the lowest NDW and SDW (Table 4). There was no significant (*p* < 0.052) interaction effect of rhizobia inoculation x site observed on bean nodulation.

**Table 4:** **Effect of rhizobia inoculation, bean variety, farm site, and their interactions on nodule dry weight, shoot dry weight, shoot nitrogen and phosphorus in the field**.

**Treatments**	**Nodule dry weight (g plant^−1^)**	**Shoot dry weight (g plant^−1^)**	**% Shoot N**	**Shoot P (ppm)**
**RHIZOBIA INOCULANT**				
ELM3	0.11 ± 0.06a	11.90 ± 0.80a	3.342 ± 0.115a	3608.60 ± 192.49a
TC	0.08 ± 0.02b	9.58 ± 0.76b	2.179 ± 0.099d	2609.20 ± 256.97c
TB	0.09 ± 0.01b	10.56 ± 0.73b	3.051 ± 0.081b	3268.80 ± 193.23ab
TCB	0.09 ± 0.02b	9.92 ± 0.78b	2.579 ± 0.057c	3120.00 ± 202.54b
TUC	0.06 ± 0.01c	7.34 ± 0.54c	1.766 ± 0.134e	3018.10 ± 241.10b
**VARIETY**				
MAC 13	0.08 ± 0.01a	9.63 ± 0.49a	2.499 ± 0.105b	2721.30 ± 116.85b
MAC 64	0.09 ± 0.01b	10.08 ± 0.49a	2.667 ± 0.089a	3528.62 ± 147.61a
**SITE**				
EUM	0.05 ± 0.01c	4.79 ± 0.23c	2.790 ± 1.01a	3335.80 ± 205.31a
ELM	0.11 ± 0.01a	13.66 ± 0.49a	2.844 ± 1.49a	2956.30 ± 210.85b
TUM	0.09 ± 0.02b	10.87 ± 0.35b	2.280 ± 0.93b	3105.70 ± 228.51ab
TLM	0.09 ± 0.01b	10.13 ± 0.38b	2.419 ± 1.06b	3102.10 ± 159.10ab
***P*****-VALUES OF THE MAIN FACTORS AND THEIR INTERACTIONS**				
Variety	0.015	0.095	0.001	< 0.001
Site	< 0.001	< 0.001	< 0.001	0.021
Rhizobia inoculant	< 0.001	< 0.001	< 0.001	< 0.001
Variety × Site	0.069	0.993	0.052	0.061
Variety × Rhizobia inoculant	0.498	0.967	0.719	0.071
Site × Rhizobia inoculant	0.052	0.217	0.010	0.051
Site × Rhizobia inoculant × Variety	0.414	0.827	0.064	0.102

*Means followed by same lower case letter(s) within the same column are not significantly different at p < 0.05 according to Tukey's HSD test. ELM3, Test native rhizobia isolate; TC, Consortium of native rhizobia; TB, Commercial inoculant (Biofix); TCB, Biofix combined with consortium; TUC, Negative control (Non-inoculated); MAC, Mid altitude climbers. ELM, Embu Lower Midland; EUM, Embu Upper Midland; TLM, Tharaka Nithi Lower Midland; TUM, Tharaka Nithi Upper Midland*.

#### Effect of rhizobia inoculation on shoot nitrogen (% N) and phosphorus (P)

**Figure 3:**
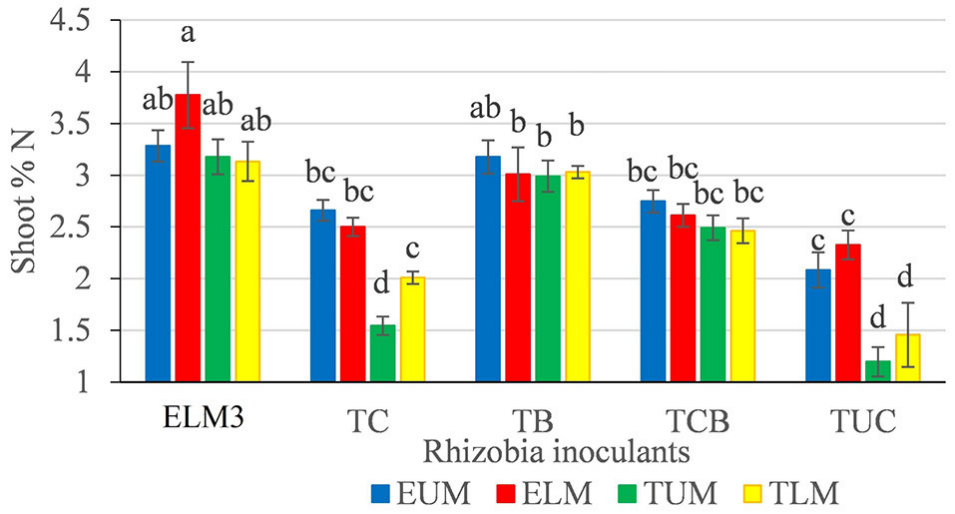
**Interactive effects of farm location with rhizobia inoculants on % shoot N of climbing beans**. Bars followed by the same letter are not significantly different according to Tukey's HSD test at *P* ≤ 0.05. ELM3, Test native rhizobia isolate; TC, Consortium of native rhizobia; TB, Commercial inoculant (Biofix); TCB, Biofix combined with consortium; TUC, Negative control (Non-inoculated); ELM, Embu Lower Midland; EUM, Embu Upper Midland; TLM, Tharaka Nithi Lower Midland; TUM, Tharaka Nithi Upper Midland.

The level of shoot nitrogen (% N) and phosphorus concentration were significantly (*p* < 0.001) enhanced by rhizobia inoculation (Table 4). Climbing beans inoculated with native isolate ELM3, had the highest shoot% N and P, while non-inoculated controls had the lowest % N and P (Table 4). Unlike in the greenhouse, there was a significant variation in % N and P (*p* = 0.001, *p* < 0.001, respectively) accumulated by the two climbing bean varieties, where MAC 64 recorded a higher shoot % N and P compared to MAC 13. Farm site significantly influenced the mean % shoot N concentration (*p* < 0.001) and shoot phosphorus (*p* = 0.021). Among the four sites, climbing beans grown in Embu County (ELM and EUM) recorded higher mean % N compared to those in Tharaka Nithi County (TLM and TUM). Interestingly, climbing beans in EUM accumulated the highest amount of P (9,335.8 ppm) despite performing poorly in nodulation. Additionally, there was a significant (*p* = 0.010) interaction effect between farm site and rhizobia inoculants on climbing bean shoot % N (Table 4). In this case, climbing beans inoculated with native isolate ELM3 accumulated a higher % shoot N in all the sites (Figure 3).

#### Effect of rhizobia inoculation on pod number (NPP) and seed yield (SY)

**Figure 4:**
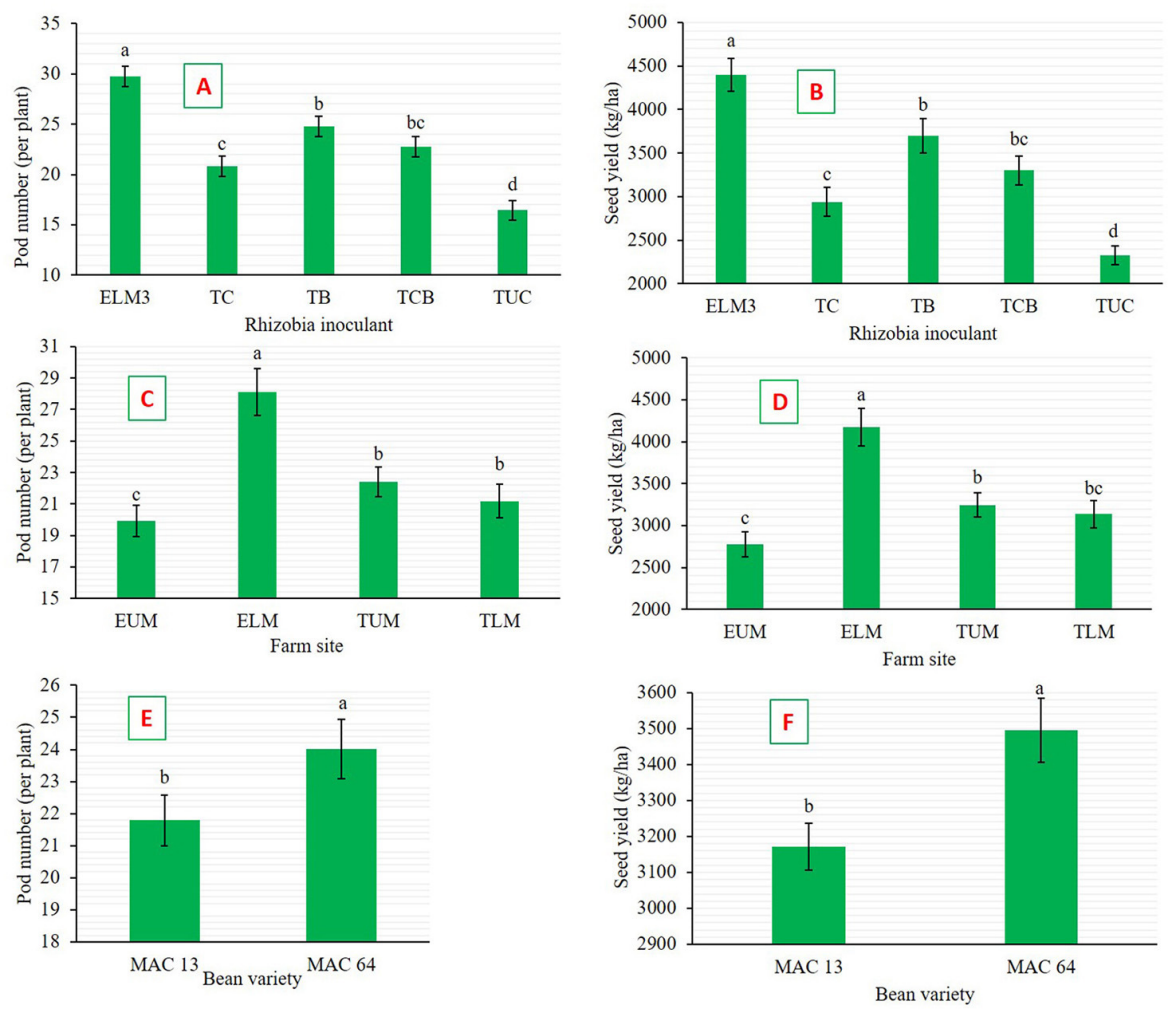
**Effects of the main factors on climbing bean yield parameters in the field. (A)** Effect of rhizobia inoculation on pod number. **(B)** Effect of rhizobia inoculation on seed yield. **(C)** Effect of farm site on pod number. **(D)** Effect of farm site on seed yield. **(E)** Effect of bean variety on pod number. **(F)** Effect of bean variety on seed yield. Bars followed by the same letter are not significantly different according to Tukey's HSD test at *P* ≤ 0.05. ELM3, Test native rhizobia isolate; TC, Consortium of native rhizobia; TB, Commercial inoculant (Biofix); TCB, Biofix combined with consortium; TUC, Negative control (Non-inoculated); ELM, Embu Lower Midland; EUM, Embu Upper Midland; TLM, Tharaka Nithi Lower Midland; TUM, Tharaka Nithi Upper Midland.

**Figure 5:**
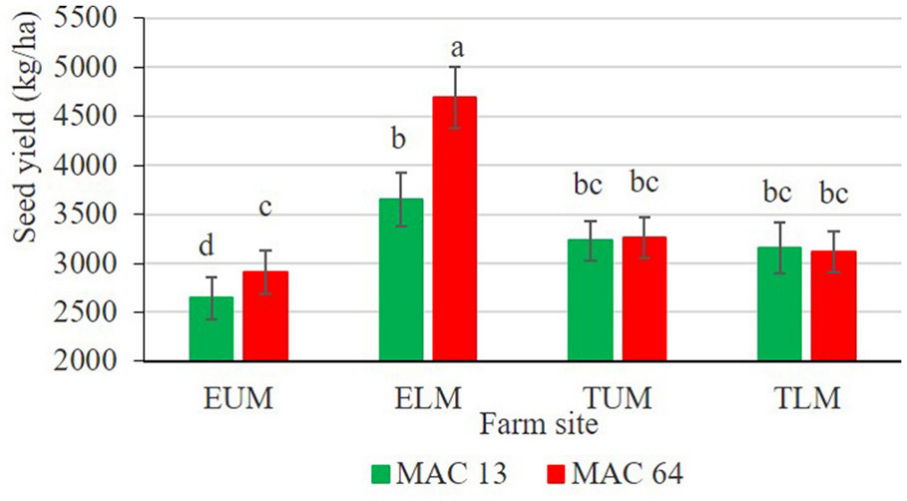
**Interactive effects of bean variety with farm site on seed yield of climbing beans**. Bars followed by the same letter are not significantly different according to Tukey's HSD test at *P* ≤ 0.05. ELM, Embu Lower Midland; EUM, Embu Upper Midland; TLM, Tharaka Nithi Lower Midland; TUM, Tharaka Nithi Upper Midland.

Rhizobia inoculation significantly enhanced (*p* < 0.001) pod number (Figure 4A) and seed yield (Figure 4B), whereby inoculated climbing beans recorded higher NPP and SY values compared to non-inoculated control. Climbing beans inoculated with native isolate ELM3 produced the highest NPP (29.75 plant^−1^) and SY (4,397.75 kg ha^−1^), while non-inoculated plants recorded the lowest NPP (16.44 plant^−1^) and SY (2,324.81 kg ha^−1^). The enhanced SY by isolate ELM3 represents 89% increase over the non-inoculated controls and 30% increase over the commercial inoculant Biofix (3,698.79 kg ha^−1^). Similarly, farm site significantly affected (*p* < 0.001) the NPP (Figure 4C) and SY (Figure 4D). Climbing beans grown in TLM and EUM agroecological zones produced the least SY. Correspondingly, bean variety significantly affected NPP (*p* = 0.001) and SY (*p* = 0.002) with MAC 64 having a higher NPP (Figure 4E) and SY (Figure 4F) compared to MAC 13 bean variety. A significant (*p* = 0.001) interaction effect was observed on SY between farm site and bean variety. In this case, MAC 64 bean variety recorded the highest seed yield (4,691.26 kg ha^−1^) in ELM while MAC 13 produced the lowest seed yield (2,644.05 kg ha^−1^) in EUM (Figure 5).

## Discussion

In the present study, the pH of soil across the farms varied in respect to the agro-ecological zonation. This could be attributed to the high mineralization rates and loss of exchangeable bases (Ca, K, and Mg) that occur through leaching in upper slopes of Mt. Kenya region (Mairura et al., 2008; Mwenda et al., 2011). Climbing beans require slightly acidic or neutral soil for growth especially when the crops depend on BNF as a source of nitrogen (Martínez-Romero, 2003). Soil pH below 5.5 and high nitrogen values above the critical limit of 0.25% described by Okalebo et al. (2002), suppresses nodulation and nitrogen fixation and does not favor the production of climbing beans (Mairura et al., 2008). The sandy-clay-loam soil texture that was recorded in ELM farm promotes soil drainage and infiltration; giving favorable conditions for bean production. A well-drained, deep, light textured soil with adequate porosity is ideal for survival and proliferation of soil bacteria, such as root-nodule rhizobia living within the plant rhizosphere (Katungi et al., 2011).

The morpho-cultural characteristics of the isolates based on Gram staining results and growth on YEMA-CR and YEMA-BTB media under dark incubation, confirmed the standard morpho-cultural characteristics of *Rhizobium* species as described by Vincent (1970), Beck et al. (1993), and Somasegaran and Hoben (1994). Morphological characteristics of rhizobia which nodulated climbing beans in the present study are similar to those reported by Kawaka et al. (2014) and Muthini et al. (2014). Temperature, pH, and soil salinity are among the abiotic factors that affect morphological characteristics of rhizobia. The isolates exhibiting a wide adaptation to environmental stresses, and are able to circumvent limiting factors and maintain a higher capacity for nitrogen fixation, could be considered for inoculum development (Berrada et al., 2012).

The greenhouse results showed that rhizobia inoculation significantly enhanced climbing bean nodulation, dry weight biomass, and symbiotic efficiency. Some of the native isolates, such as ELM3 showed superior performance in all the tested symbiotic parameters compared to the commercial inoculant Biofix and non-inoculated control. The enhanced performance showed by some of the native isolates could be attributed to their ability to infect, nodulate and fix nitrogen with MAC 13 and MAC 64 climbing beans. On the other hand, the performance of commercial inoculant is largely dependent on not only the number of viable rhizobia cells but also the presence or absence of other microbial contaminants (Aliyu et al., 2013). These findings are consistent with the previous findings (Onyango et al., 2015), which showed the competitive potential of Bambara native isolates from Western Kenya when compared to the commercial strain USDA 110. Most of the root nodules from our study showed pink coloration, indicating the presence of iron-containing protein required for effective nitrogen fixation (Farid and Navabi, 2015). Pink nodules are known to contain and actively express *nifH* genes that codes for the synthesis of nitrogenase enzymes responsible for the reduction of N to NH_3_ (Rondon et al., 2007). Between the two climbing bean varieties, MAC 64 showed superior nodulation and biomass accumulation over MAC 13 under controlled greenhouse conditions. The high nodule dry matter recorded by MAC 64 reflects a more efficient symbiotic nitrogen fixation that could result to an increased shoot biomass. These results are consistent with the observations made by Gicharu et al. (2013), who noted the differences in nodulation and biomass accumulation among three climbing bean cultivars (G59/1-2, NG224-4, and Cargamanto) grown under controlled conditions in the greenhouse. Our findings may, therefore, suggest the superior performance of MAC 64 over MAC 13 climbing bean varieties in the greenhouse environment.

The plant SDW was used during the study to estimate the symbiotic nitrogen-fixing efficiency of the native rhizobia isolates (Gibson, 1987; Beck et al., 1993). This method is easy to use and it is relatively cheap; and most appropriate for use in soils with low nitrogen content (Rondon et al., 2007). Symbiotic efficiency (SEF) differed significantly among the isolates tested in the greenhouse. In our study, all the native isolates that nodulated had higher SEF, which ranged from 86.7 to 123.72%. Such findings suggest that native isolates enhanced nitrogen fixation, which consequently increased SDW and nitrogen accumulation. These results concur with the findings of Kawaka et al. (2014) who reported SEF ranging between 67 and 170% when common beans were inoculated with native rhizobia in Western Kenya. Similarly, Mungai and Karubiu (2011) reported that native rhizobia isolated from common beans from Njoro, Kenya, had higher SEF compared to the commonly used commercial inoculants Biofix and USDA 9030. Based on the rating scale used by Lalande et al. (1990), it was evident from our study that native isolates ELM3, ELM4, ELM5, ELM8, and ELM9 were highly effective (SEF > 80%) in symbiotic nitrogen fixing efficiencies. The consortium of native isolates had the lowest SEF, inferring that the combination of several native isolates could not provide any functional advantage compared to single isolates (Meghvansi et al., 2010).

The significant correlation between nodule dry weight and shoot dry weight in the greenhouse experiments confirmed the dependence of shoot biomass on nodulation (Kawaka et al., 2014). In addition, the results from this study support the assertion made by Delić et al. (2010), that there is a direct relationship between nodulation and nitrogen accumulation in legumes. Our findings, therefore, demonstrated that rhizobia inoculation enhanced nodulation and nitrogen fixation, which improved shoot nitrogen nutrition and plant biomass. These results concur with the findings of Unkovich et al. (2010) who reported a strong positive correlation between shoot biomass and nitrogen accumulated by rhizobia-inoculated lentils and peas. This study supports the use of such parameters as measures of nitrogen fixation potential of rhizobia isolates in symbiosis with legumes (Patra et al., 2012).

Inoculation of climbing beans in the field significantly enhanced nodule and shoot dry weights, pod number and seed yields of MAC 13 and MAC 64 climbing beans. In our study, the superiority of native isolate ELM3 over other inoculants, in respect to nodulation, biomass accumulation and yield, indicate the existence of effective rhizobia isolates in the soil of Eastern Kenya. Native strains are more competitive in nodule infection and occupancy compared to commercial inoculants because they are well adapted to the local agro-climatic conditions (Meghvansi et al., 2010). In addition, native rhizobia strains interact positively with the resident microbial populations resulting to improved soil health, nutrient availability and enhanced yields (Nkot et al., 2015; Tena et al., 2016). Rhizobia interact positively with other microbial inoculants, such as arbuscular mycorrhizal fungi resulting to improved soil health, nutrient availability and enhanced crop yields (Meng et al., 2015; Oruru and Njeru, 2016), therefore showing its importance in sustainable agricultural farming practices.

According to Morad et al. (2013), inoculated beans showed higher nodulation, pod number and seed yield compared to the control plants, which was in line with the findings of our study. Interestingly, the individual performance of native isolates in the field was better than that of the commercial inoculants. Our findings are further supported by Tena et al. (2016) who concluded that native rhizobia isolates in the field often out-compete the commercial rhizobia inoculant, highlighting the potential of native isolates in bean production. Nonetheless, commercial rhizobial inoculants have potential to enhance growth and nitrogen fixation of legumes (Thuita et al., 2011; Ulzen et al., 2016). In this study, the average performance of Biofix in nitrogen fixation could be attributed to the soil properties and unfavorable agroecological conditions in Eastern Kenya, which affects the rhizobia-legume interaction within the rhizosphere. Meghvansi et al. (2010) further stress the importance of using effective native rhizobia, which are adapted to the local soil and environmental conditions as biofertilizer inoculants in bean production.

Inoculation of climbing beans with native consortium isolates and the combination of native consortium + Biofix had no significant effect on NDW and SDW. This shows that diversifying rhizobia isolates in our field study had no beneficial advantage over the use of single rhizobia isolates. Martínez-Romero (2003) noted that bean inoculation with a diverse rhizobia population does not necessarily translate to a higher legume grain yield due to the inability of some rhizobia strains to cause nodulation and affect nitrogen fixation. In addition, Nkot et al. (2015) added that the establishment, persistence, and effectiveness of an introduced rhizobia strain often decrease with increase in population density due to the possibility of negative microbial interaction or incompatibility with the other symbionts within the rhizosphere.

In this study, there was a high percentage shoot N and P recorded in climbing beans inoculated with native isolate ELM3 in all the four agroecological sites. This demonstrated the consistency and superiority of the native isolate in nitrogen fixation both in the greenhouse and in the field conditions. Phosphorus is an essential nutrient that drives BNF, therefore, P-deficient soils could result into low BNF despite the high abundance of native rhizobia strains (Unkovich et al., 2010). In our study, phosphorus was applied in form of (TSP) fertilizers at a rate of 46 kg P_2_O_5_ ha^−1^ to achieve maximum nitrogen fixation (Gicharu et al., 2013). The significant interactive effects between bean variety and farm site on seed yield indicate that the two bean varieties responded differently in the four agroecological zones of Eastern Kenya. The MAC 64 climbing beans grown in Embu lower midland agroecological zone produced the highest climbing bean seed yield. This could be attributed to various factors including favorable soil pH, the soil type, effectiveness of rhizobia and climatic conditions (Rondon et al., 2007). Monyo and Laxmipathi (2014) reported that different soybean and common bean varieties are suitable for production in specific agroecological zones of Malawi due to the varying ecological and climatic conditions.

This study has proved the superiority of native rhizobia over introduced inoculants in four different agroecological zones with no history of previous inoculation. Thus, the study recognizes the sense of the “geographical place factor” for microorganisms to adapt to different physical and biological properties of an agricultural ecosystem. The use of native rhizobia in supplementing nitrogen-based chemical fertilizers in legume production is of great significance as it promotes local self-management of natural agroecosystems for legume production (Meghvansi et al., 2010). According to Nkot et al. (2015), the use of native rhizobia as bio-fertilizers would enhance soil biodiversity conservation, because bio-fertilization limits the adverse negative effects brought about by the inorganic fertilizers on below-ground biodiversity. The effective use of locally available rhizobia strains, therefore, promotes the delivery of sustainable agroecosystem services (Singh et al., 2016) in legume production.

## Conclusion

Generally, the current study demonstrated the presence of effective native rhizobia that are potentially superior compared to the available commercial inoculant (Biofix) in nodulation, symbiotic efficiency and yield performance of MAC 13 and MAC 64 climbing bean varieties. There exists a significant variation in symbiotic efficiencies of native rhizobia isolates nodulating with MAC 13 and MAC 64 climbing beans. The native isolate ELM3 had the highest symbiotic efficiency compared to other isolates tested in the greenhouse. Consistencies in the performance isolate ELM3 in the greenhouse as well as in all the four agroecological zones of Eastern Kenya clearly indicate its symbiotic superiority and adaptability to the region. The low efficiency of the combination of Biofix + a consortium of native isolates indicates that diversifying rhizobia isolates in the field had no additive effect over the use of independent isolates. Based on the superior response of MAC 64 climbing bean variety toward rhizobia inoculation, as evident in the greenhouse and field experiments, further breeding of this variety should be considered. Moreover, farmers should adopt the use of effective native rhizobia inoculants to enhance climbing bean production in the region. Further experiments should focus on genetic characterization and large-scale multiplication of the effective native isolate ELM3 before being recommended as a bean rhizobia inoculant.

## Author contributions

JK, OO, JM, and EN conceived and designed the research and data collection tools and participated in drafting the manuscript. GK and SM collected the data, participated in data analyses and wrote the manuscript. EN performed data analyses. All authors read and approved the final manuscript.

## Funding

This work was supported by the Regional Universities Forum for Capacity Building in Agriculture (RUFORUM) 5th Graduate Research Grant (GRG), Grant No. RU 2014 GRG-102.

### Conflict of interest statement

The authors declare that the research was conducted in the absence of any commercial or financial relationships that could be construed as a potential conflict of interest.

## Abbreviations

AEZ: Agroecological zones
ELM: Embu Lower Midland
EUM: Embu Upper Midland
MAC: Mid-altitude climbers
SEF: Symbiotic efficiency
TLM: Tharaka Nithi Lower Midland
TUM: Tharaka Nithi Upper Midland.
